# Prognostic Impact of Cachexia Index in Patients Undergoing Surgery for Esophageal Cancer

**DOI:** 10.1245/s10434-026-19219-7

**Published:** 2026-02-09

**Authors:** Tomoki Kaname, Kotaro Sugawara, Koichi Yagi, Shoh Yajima, Yoshiyuki Miwa, Shuichiro Oya, Asami Okamoto, Raito Asaoka, Haruki Kojima, Yoshifumi Baba

**Affiliations:** Department of Gastrointestinal Surgery, Graduate School of Medicine, The University of Tokyo, Tokyo, Japan

**Keywords:** Cachexia, Disease-free survival, Esophageal neoplasms, Esophagectomy, Nutritional status, Overall survival, Sarcopenia

## Abstract

**Background:**

Cachexia index (CXI) is a recently proposed biomarker reflecting the cachectic condition, including inflammation, sarcopenia and nutritional status, of patients with various malignancies. We evaluated its prognostic impact in patients undergoing esophagectomy for esophageal cancer (EC).

**Methods:**

A total of 378 patients were retrospectively reviewed. CXI was calculated as skeletal muscle index × serum albumin level/neutrophil-to-lymphocyte ratio. The sex-specific lowest quartile defined the Low-CXI group. Univariate and multivariate Cox proportional hazards models were applied to identify independent prognostic factors for overall survival (OS) and disease-free survival (DFS).

**Results:**

The low-CXI group was significantly associated with older age (*P* < 0.001), more severe comorbidity (*P* = 0.001), and more advanced pathological stage (*P* = 0.032). Patients in the low-CXI group had poorer OS and DFS than those in the high-CXI group (both *P* < 0.001). Multivariate analysis revealed that low-CXI was independently associated with poor OS (*P* = 0.002) and DFS (*P* = 0.002). In the cause-specific survival analyses, low-CXI was a predictor of deaths from non-EC-related causes. Subdivision into pathological stage (pStage) 0–I and II/III revealed that significant survival differences according to CXI were observed in both pStage 0–I (*P* = 0.045 for OS, and 0.029 for DFS) and pStage II/III (*P* < 0.001 for OS, and 0.002 for DFS) patients.

**Conclusions:**

Low-CXI was independently associated with poor OS and DFS in patients undergoing surgery for EC, in both early- and advanced-stage patients. Furthermore, CXI may also be useful for predicting non-EC-related mortality.

**Supplementary Information:**

The online version contains supplementary material available at 10.1245/s10434-026-19219-7.

Esophageal cancer (EC) is a life-threatening disease, accounting for approximately 445,000 deaths worldwide each year.^1^ Despite recent advances in multimodal therapy including surgery, chemotherapy and radiotherapy, the survival outcomes of patients with EC remain poor.^2,3^

In recent years, cancer cachexia has attracted attention as an underlying condition associated with poor prognosis.^4,5^ Cachexia is defined as a condition characterized by an ongoing loss of skeletal muscle mass that cannot be fully reversed by conventional nutritional therapy.^6^ Cancer cachexia is reportedly associated with increased mortality, a higher rate of surgical complications and greater chemotherapy-related toxicity, resulting in poor outcomes in patients with malignancies.^7^ In particular, esophageal cancer often involves poor oral intake due to bowel obstruction and dysphagia, both of which further aggravate malnutrition.^2,8–12^

Cachexia index (CXI) is an emerging biomarker for evaluating the severity of cachexia.^13^ It is calculated on the basis of skeletal muscle index (SMI), serum albumin level, and neutrophil-to-lymphocyte ratio (NLR). Low-CXI is reportedly associated with poor overall survival (OS) and disease-free survival (DFS) across several types of malignancies.^14–18^ Only a few studies have investigated the prognostic impacts of CXI in patients with esophageal cancer;^19,20^ therefore, the survival and oncological impacts of CXI remain to be fully addressed. In this study, we investigated the survival impacts of CXI in patients who underwent surgery for EC.

## Patients and Methods

### Patients

A total of 480 patients with esophageal cancer underwent esophagectomy at the University of Tokyo Hospital between 2006 and 2016. After excluding 44 patients who underwent salvage esophagectomy, 6 who underwent pharyngolaryngeal esophagectomy, 6 with melanoma or neuroendocrine carcinoma, 6 who died within 90 days after surgery, 40 lacking appropriate clinical data, 378 patients were included. Tumor stage was determined according to the UICC 7^th^ edition. Patients with pathological stage IV cancer, defined by the presence of distant metastasis, were excluded from the analysis because their treatment strategies were substantially different from those for patients with stage III or lower disease and the number of such patients was small during the study period.^21^ The final follow-up was conducted in June 2025 and the median follow-up duration was 109.5 months among survivors. This retrospective study was approved by the Ethics Committee of the Faculty of Medicine, the University of Tokyo (Approval ID: 3962).

### Surgical Treatments

All patients underwent subtotal esophagectomy by either the Ivor Lewis or McKeown procedure, as previously reported.^10,22^ The surgical approaches included open right thoracotomy, video-assisted thoracoscopic surgery (VATS), and robot-assisted transmediastinal esophagectomy (TME).^23^ Three-field lymphadenectomy was generally applied for upper and middle thoracic esophageal cancer, whereas two-field lymphadenectomy was applied for lower thoracic and abdominal esophageal cancer.^24^

### Measurement of CXI

The skeletal muscle index (SMI) was calculated from preoperative computed tomography (CT) images. The total cross-sectional area of skeletal muscle (cm^2^) was measured at the level of the third lumbar vertebra using OsiriX version 9.5 (Newton Graphics, Inc., Sapporo, Japan), an open-source software for medical image analysis.^25^ Skeletal muscle was identified and quantified on the basis of Hounsfield unit thresholds (−29 to +150). SMI (cm^2^ / m^2^) was calculated by dividing the total skeletal muscle at the third lumbar vertebra (cm^2^) by the square of the patient’s height (m^2^). The neutrophil-to-lymphocyte ratio (NLR) was obtained by dividing the absolute neutrophil count by the absolute lymphocyte count. The cachexia index (CXI) was then calculated using the following formula: CXI = (SMI × serum albumin level [g/dL]) / NLR.^13^ Patients were dichotomized into the low- and high-CXI groups according to the lowest sex-specific quartile of CXI in our cohort. The discriminatory ability of CXI and its components, including SMI, NLR, and serum albumin level (ALB), was evaluated using the concordance index (C-index).^10,26^

### Statistical Analysis

Continuous variables were analyzed using the Wilcoxon rank-sum test, and categorical variables were analyzed using the *χ*^2^ test. Overall survival (OS), disease-free survival (DFS), and recurrence-free survival (RFS) were calculated from the date of surgery. In this study, DFS is defined with both death and recurrence as events, whereas RFS was defined with recurrence alone as the event. Survival curves were estimated using the Kaplan–Meier method, and statistical differences were assessed with the log-rank test. Univariate and multivariate analyses were conducted using Cox proportional hazards models to identify independent prognostic factors. Two-sided *P*-values < 0.05 were considered statistically significant. The C-index was calculated using R (version 4.5.1, The R Foundation for Statistical Computing) and all other statistical analyses were performed using JMP software (version 18.1.1, SAS Institute, Cary, NC).

## Results

### Patient Characteristics According to CXI

The median age of the patients was 66 years (interquartile range: 60–72). The high-CXI group included 284 patients, and the low-CXI group included 94 patients. Table 1 summarizes the patient background according to CXI.

**Table 1:** Characteristics of 378 patients

Variables	Low CXI (*n* = 94)	High CXI (*n* = 284)	*P* value
Age, Median (range)	69(46–92)	65(39–86)	< 0.001 *
Sex			0.95
Male	80(85.11%)	241(84.86%)	
Female	14(14.89%)	43(15.14%)	
Comorbidity (CCI ≥ 2)	36(38.30%)	58(20.42%)	0.001*
Tissue Type			0.19
SCC	88(93.62%)	275(96.83%)	
AC	2(2.13%)	6(2.11%)	
Others	4(4.26%)	3(1.06%)	
Location			0.90
Ce-Ut	14(14.89%)	39(13.73%)	
Mt	45(47.87%)	132(46.48%)	
Lt-Ae	35(37.23%)	113(39.79%)	
Neoadjuvant chemotherapy	16(17.02%)	15(5.28%)	< 0.001*
Surgery			0.37
TTE	77(81.91%)	211(74.30%)	
TME	10(10.64%)	47(16.55%)	
VATS	5(5.32%)	22(7.75%)	
THE	2(2.13%)	4(1.41%)	
Complications (≥ Grade II ^†^)	52(55.32%)	147(51.76%)	0.55
Pneumonia	21(22.3%)	56(19.72%)	0.59
Anastomotic leakage	16(17.0%)	57(20.1%)	0.51
pStage (UICC 7th)			0.008*
Stage 0-I	26(27.66%)	113(39.79%)	
Stage II	16(17.02%)	66(23.24%)	
Stage III	52(55.32%)	105(36.97%)	

^*^Statistically significant (*P* < 0.05)

^†^Clavien–Dindo classification

CXI, cachexia index; CCI, Charlson comorbidity index; SCC, squamous cell carcinoma; AC, adenocarcinoma; Ce, cervical esophagus, Ut, upper thoracic esophagus; Mt, middle thoracic esophagus, Lt, lower thoracic esophagus; Ae, abdominal esophagus; TTE, transthoracic esophagectomy; TME, transmediastinal esophagectomy; VATS, video-assisted thoracic surgery, THE, transhiatal esophagectomy, pStage, pathological stage

Age was significantly higher in patients in the low-CXI group than those in the high-CXI group (median 69 versus 65 years, *P* < 0.001). The proportion of patients with a Charlson Comorbidity Index (CCI) ≥ 2 was significantly higher in the low-CXI group than in the high-CXI group (38.3% versus 20.4%, *P* = 0.001). The low-CXI group had a significantly higher proportion of patients with pathological stage ≥ II (72.3% versus 60.2%, *P* = 0.032) and of those who received neoadjuvant chemotherapy (17.0% versus 5.3%, *P* < 0.001). There were no significant differences in histological type, tumor location, or surgical procedure between the two groups.

### Survival Outcomes According to CXI

**Fig. 1:**
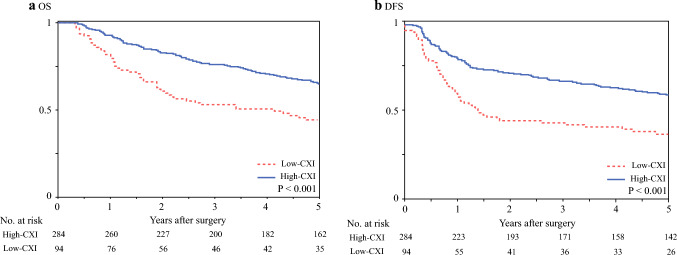
Survival outcomes according to cachexia index (CXI). Patients in the low-CXI group had **a** significantly poorer overall survival (OS) (*P* < 0.001) and **b** significantly poorer disease-free survival (DFS) (*P* < 0.001) than those in the high-CXI group

**Fig. 2:**
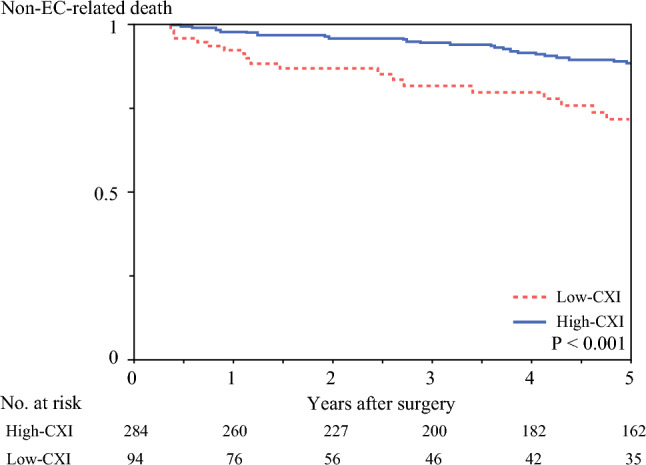
Non-esophageal cancer (EC)-related mortality according to cachexia index (CXI). Patients in the low-CXI group had significantly higher non-EC-related mortality (*P* < 0.001)

Patients in the low-CXI group had significantly worse OS (5-year OS: 44.2% versus 65.0%; *P* < 0.001), DFS (5-year DFS: 33.0% versus 56.5%; *P* < 0.001), and RFS (5-year RFS: 51.4% versus 68.0%; *P* = 0.002) than those in the high-CXI group (Fig. 1, Supplementary Fig. 1). Figure 2 shows that deaths due to non-EC-related diseases were more prevalent in low-CXI patients than in high-CXI patients (*P* < 0.001), and this association remained unchanged after inclusion of the six patients who died within 90 days after surgery (*P* < 0.001). Together, low-CXI was significantly associated with both oncological and nononcological outcomes in our cohort.

### Low-CXI was Independently Associated with Poor OS and DFS

In the univariate analysis, low-CXI (HR 2.01, 95% CI: 1.46–2.77, *P* < 0.001), older age (*P* = 0.006), CCI ≥ 2 (*P* < 0.001), and pathological stage III (compared with stage 0 or I, *P* < 0.001) were significantly associated with OS (Table 2).

**Table 2.** Cox hazards model for overall survival

Variables	Univariable analysis			Multivariable analysis		
	HR	95% CI	*P* value	HR	95% CI	*P* value
Age > 65	1.54	1.13–2.10	0.006*	1.27	0.92–1.75	0.15
Male	1.46	0.93–2.31	0.10			
CCI ≥ 2 (versus 0–1)	1.83	1.33–2.51	< 0.001*	1.65	1.18–2.30	0.004*
pStage 0-I	Ref			Ref		
pStage II	1.04	0.66–1.63	0.86	1.08	0.68–1.70	0.75
pStage III	2.63	1.86–3.73	< 0.001*	2.53	1.78–3.61	< 0.001*
Locus (Ce-Ut versus Mt-Lt-Ae)	1.20	0.80–1.80	0.37			
Complications (≥ Grade II ^†^)	1.18	0.87–1.59	0.28			
CXI (Low versus High)	2.01	1.46–2.77	< 0.001*	1.56	1.12–2.18	0.009*

^*^Statistically significant (*P* < 0.05)

^†^Clavien–Dindo classification

CCI, Charlson Comorbidity Index; pStage, pathological stage; Ce, cervical esophagus; Ut, upper thoracic esophagus; Mt, middle thoracic esophagus, Lt, lower thoracic esophagus; Ae, abdominal esophagus; CXI, cachexia index

In the subsequent multivariate analysis, low-CXI (HR 1.56, 95% CI: 1.12–2.18, *P* = 0.009), CCI ≥ 2 (*P* = 0.004), and pathological stage ≥ III (versus pStage 0–I, *P* < 0.001) were identified as independent factors for predicting poor OS. Regarding DFS, low-CXI, older age, CCI ≥ 2, and pathological stage III also demonstrated significant associations (Table 3). Multivariate analysis revealed that low-CXI (HR 1.55, 95% CI: 1.14–2.12, *P* = 0.006), CCI ≥ 2 (*P* = 0.043), and pathological stage ≥ III (versus pStage 0-I, *P* < 0.001) were independent factors for predicting poor DFS.

**Table 3.** Cox hazards model for disease-free survival

Variables	Univariable analysis			Multivariable analysis		
	HR	95% CI	*P* value	HR	95% CI	*P* value
Age > 65	1.39	1.05–1.84	0.022*	1.18	0.88–1.58	0.28
Male	1.14	0.77–1.70	0.50			
CCI ≥ 2 (versus 0–1)	1.51	1.12–2.04	0.007*	1.39	1.01–1.91	0.043*
pStage 0–I	Ref			Ref		
pStage II	1.13	0.74–1.72	0.58	1.14	0.75–1.75	0.53
pStage III	3.28	2.37–4.54	<0.001*	3.17	2.28–4.41	< 0.001*
Locus (Ce-Ut versus Mt-Lt-Ae)	1.16	0.80–1.69	0.43			
Complications (≥ Grade II^†^)	1.17	0.89–1.54	0.27			
CXI (Low versus High)	1.95	1.45–2.62	<0.001*	1.55	1.14–2.12	0.006*

^*^Statistically significant (*P* < 0.05)

^†^Clavien–Dindo classification

CCI, Charlson Comorbidity Index; pStage, pathological stage; Ce, cervical esophagus; Ut, upper thoracic esophagus; Mt, middle thoracic esophagus; Lt, lower thoracic esophagus; Ae, abdominal esophagus; CXI, cachexia index

The C-index of CXI was 0.591, which was higher than those of its components: 0.583 for ALB, 0.563 for NLR, 0.567 for SMI.

### Stage-Based Subgroup Analysis

**Fig. 3:**
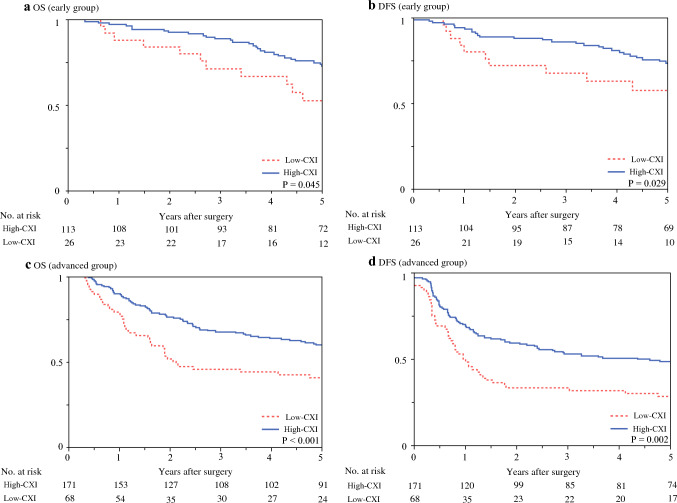
Survival outcomes of early- and advanced-stage patients according to cachexia index (CXI). In the early-stage subgroup, **a** overall survival (OS) and **b** disease-free survival (DFS) curves were well demarcated according to CXI (*P* = 0.045 and* P* = 0.029, respectively). In the advanced-stage subgroup, **c** OS and **d** DFS were significantly classified according to CXI (*P* < 0.001 and *P* = 0.002, respectively)

Lastly, we conducted a subgroup analysis, subdividing our cohort into patients with early-stage cancer (pathological stage 0–I) and those with advanced-stage cancer (pathological stage II or higher). This subgroup analysis revealed that patients in the low-CXI group had poorer OS than those in the high-CXI group in both early- and advanced-stage subgroups, with 5-year survival rates of 52.5% versus 72.8% (*P* = 0.045) and 40.8% versus 59.9% (*P* < 0.001), respectively (Fig. 3). Similarly, DFS was significantly worse in the low-CXI group than in the high-CXI group in both stage categories: 57.7% versus 73.6% for early-stage subgroup (*P* = 0.029) and 28.4% versus 48.6% for advanced-stage subgroup (*P* = 0.002).

## Discussion

Our study demonstrated that patients with esophageal cancer (EC) with low CXI had significantly poorer survival outcomes than those with high CXI, including OS, DFS, and RFS. Further, low CXI was associated with high incidence of deaths due to non-EC-related diseases. In the subgroup analysis, low CXI showed significant impacts on survival in both early- and advanced-stage patients with EC. The C-index of CXI was higher than that of each individual component. These findings suggest the robust survival impact of CXI in patients undergoing surgery for EC.

Patient characteristics significantly differed between the two groups. Of note, low CXI was associated with older age, the presence of severe comorbidities, and advanced tumor stage. CXI is calculated on the basis of SMI, NLR, and serum albumin levels, all of which have been reported to be associated with indicators of frailty, such as age and CCI.^27–30^ As for disease stage, the association between low CXI and advanced stage has been reported in various malignancies,^16,17,28,29^ which is in line with our findings. Overall, CXI reflects both physiological and oncological backgrounds of patients with EC.

Although recent studies have revealed low CXI to be associated with poor OS in various malignancies including gastroesophageal cancers,^14–17,20^ the impact of CXI on DFS of patients with esophageal cancer remains to be clarified. In the present study, low CXI was independently associated not only with poor OS but also with poor DFS. Recent studies have demonstrated similar oncological impacts of CXI status in gastrointestinal cancers.^28,31,32^ Taken together, these findings suggest that CXI might have the potential to reflect the biological aggressiveness of cancers, including both shortened survival and increased recurrence risk, which cannot be fully assessed by cancer stage alone.

Furthermore, our cohort included a large number of patients with esophageal squamous cell carcinoma (ESCC), nearly 90%, although a recent study showed the significance of CXI in a cohort mainly composed of patients with adenocarcinoma.^20^ Body composition greatly differs between patients with ESCC and those with esophageal adenocarcinoma.^33^ Overall, our study is the first to demonstrate the survival and oncological impacts of low-CXI in patients with ESCC.

It is noteworthy that significant differences in OS and DFS according to the CXI status were found both in the early- and advanced-stage groups. Previous studies have suggested the survival impacts of each host-related nutritional and physiological status alone to be diminished in advanced-stage cancer patients.^34–37^ In contrast, the combination of multiple factors—particularly nutritional and inflammatory factors—has demonstrated prognostic significance across all cancer stages in gastrointestinal malignancies.^38–41^ These results highlight the utility of composite markers that integrate multiple nutritional, inflammatory, and physiological factors, such as CXI. The higher C-index of CXI compared with those of its individual components supports this concept and suggests its utility.

Not only ESCC-related deaths, but also mortality from non-ESCC-related diseases was higher in low-CXI patients than in high-CXI patients. This finding is reasonable given that CXI reflects nutritional, inflammatory, and sarcopenic status, which reportedly affects overall health status and general mortality.^42,43^ CXI may be useful for identifying frail patients with ESCC, which might explain the significant prognostic influence of CXI in patients with early-stage ESCC.

Overall, our results suggest that CXI, a newly-proposed biomarker, is useful for predicting long-term survival and oncological outcomes of patients with ESCC irrespective of cancer stage. Of note, low CXI was independently associated with poor OS and poor DFS even after adjusting for other possible predictors such as advanced age and comorbidity. CXI can be easily calculated from CT and blood test examinations, which are routinely performed before surgery in clinical practice. The present study suggests that CXI, a novel composite marker, may have stronger discriminatory ability for prognosis than its individual components, such as SMI. On the other hand, the calculation of CXI is relatively complex, and it cannot be regarded as a simple biomarker compared with its components. Therefore, the clinical utility of CXI should be carefully evaluated from multiple perspectives in future studies.

Treatment strategies for patients with sarcopenia and inflammation remain to be established. Early identification of myopenia and myosteatosis employing CT scans and systemic inflammatory response biomarkers may allow early therapeutic intervention. Prior investigations suggested that multimodal therapy combining nutritional support, physical exercise, and pharmacological interventions moderates the host systemic inflammatory response and prevents cancer patients from developing refractory cachexia.^44–46^ Our observations suggested that CXI may be useful for optimizing perioperative management.

Our study has several limitations. First, our cohort included both patients who received neoadjuvant chemotherapy (NAC) and those who underwent upfront surgery. NAC reportedly changes the nutritional, inflammatory, and sarcopenic status of patients.^47–49^ Second, the optimal threshold for CXI remains to be determined. In this study, we set the cut-off value as the sex-specific lowest quartile to focus particularly on patients with low CXI. However, previous studies have employed various methods, such as receiver operating characteristic curve analysis, median values, or specific cut-off points.^13–17,20,29,50,51^ Although this flexibility may reflect the prognostic utility of CXI, a standardized threshold is needed for clinical use. Third, patients with esophageal squamous cell carcinoma are known to have a higher prevalence of underweight status compared with those with esophageal adenocarcinoma, which is common in Western countries.^33^ This difference may influence the applicability of our findings to Western populations. Finally, this was a retrospective study conducted at a single institution. To validate our findings, further large-scale multicenter studies are warranted.

## Supplementary Information

Below is the link to the electronic supplementary material.

Supplementary file1 (DOCX 136 kb)
