# Supplementary material for: Prognostic Impact of Cachexia Index in Patients Undergoing Surgery for Esophageal Cancer
Source: Ann Surg Oncol. 2026 Feb 9;33(6):5339–47. doi: 10.1245/s10434-026-19219-7 (PMC13179219; doi:10.1245/s10434-026-19219-7)
Supplement: Supplementary file 1 — Supplementary file1 (DOCX 136 kb) [file 10434_2026_19219_MOESM1_ESM.docx]

**Figure Legends**

**
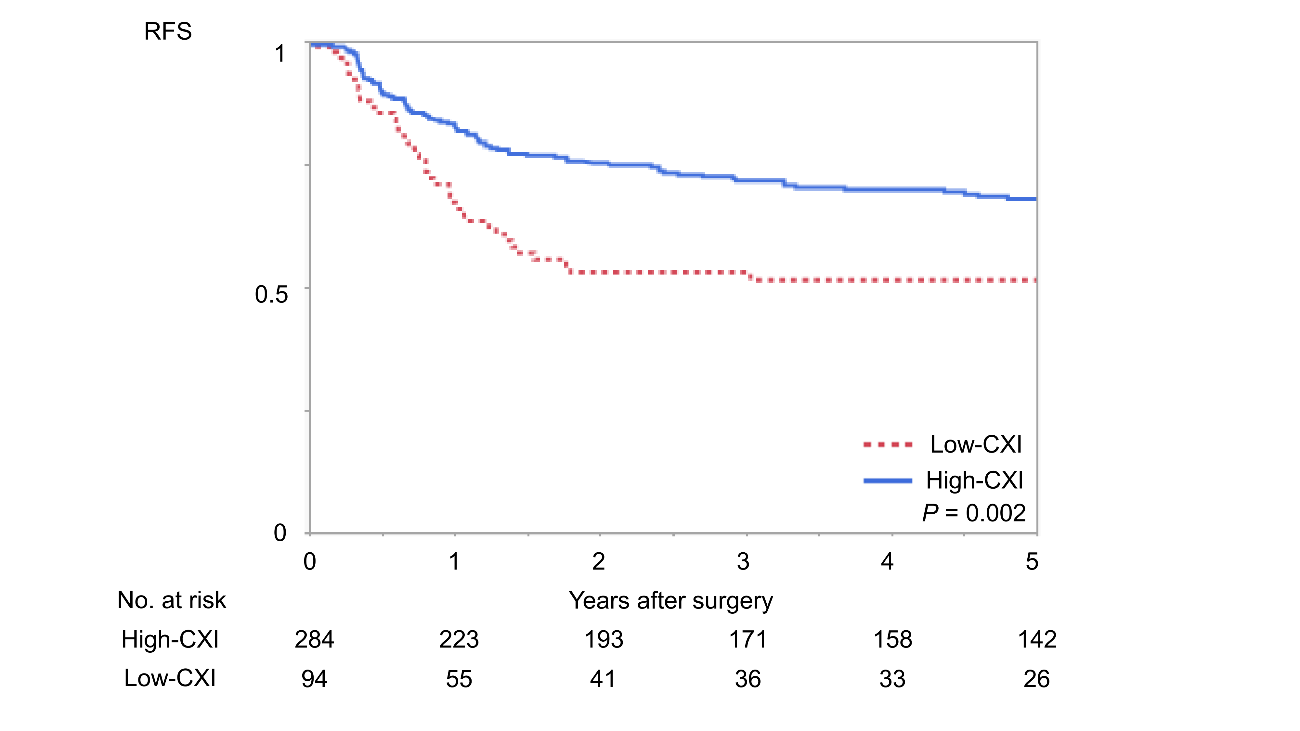
**

Supplementary Fig. 1 Patients in the Low-CXI group had significantly poorer recurrence-free survival (RFS) (*P =* 0.002) than those in the High-CXI group.
